# Multifunctional Superamphiphobic Coating Based on Fluorinated TiO_2_ toward Effective Anti-Corrosion

**DOI:** 10.3390/ma17102203

**Published:** 2024-05-08

**Authors:** Xiao Huang, Xinghua Gao, Xin Wang, Hongfei Shang, Shujun Zhou

**Affiliations:** 1 School of Mechanical and Electrical Engineering, China University of Mining and Technology (Beijing), Beijing 100083, China; hx@cumtb.edu.cn (X.H.); 17810286684@sina.cn (X.G.); 2 Surface Engineering Institution, AECC Beijing Institute of Aeronautical Materials, Beijing 100095, China; rasheed990918@163.com; 3 State Key Laboratory of Tribology, Tsinghua University, Beijing 100084, China; shanghongfei@tsinghua.edu.cn

**Keywords:** superamphiphobic, armor structure, spraying method, robust, multifunctional

## Abstract

The application of superamphiphobic coatings improves the surface’s ability to repel fluids, thereby greatly enhancing its various functions, including anti-fouling, anti-corrosion, anti-icing, anti-bacterial, and self-cleaning properties. This maximizes the material’s potential for industrial applications. This work utilized the agglomeration phenomenon exhibited by nano-spherical titanium dioxide (TiO_2_) particles to fabricate 1H,1H,2H,2H-perfluorodecyltriethoxysilane (PFDTES) modified TiO_2_ (TiO_2_@fluoroPOS) fillers with low surface energy. This was achieved through the in-situ formation of protective armor on the surface of the agglomerates using the sol-gel method and fluorination modification. Polyvinylidene fluoride-tetrafluoropropylene (PVDF-HFP) and TiO_2_@fluoroPOS fillers were combined using a spraying technique to prepare P/TiO_2_@fluoroPOS coatings with superamphiphobicity. Relying on the abundance of papillae, micropores, and other tiny spaces on the surface, the coating can capture a stable air film and reject a variety of liquids. When the coatings were immersed in solutions of 2 mol/L HCl, NaCl, and NaOH for a duration of 12 h, they retained their exceptional superamphiphobic properties. Owing to the combined influence of the armor structure and the organic binder, the coating exhibited good liquid repellency during water jetting and sandpaper abrasion tests. Furthermore, the coating has shown exceptional efficacy in terms of its ability to be anti-icing, anti-waxing, and self-cleaning.

## 1. Introduction

By improving a surface’s ability to repel fluids, special anti-wetting coatings can maximize the material’s potential for industrial applications by improving the surface’s anti-fouling [1,2,3], anti-corrosion [3,4,5,6], anti-icing [6,7,8,9,10], anti-bacterial [7], and many other properties [11]. Researchers have used low surface energy materials to construct micro-nano roughness structures in order to prepare superhydrophobic coatings, which are widely used in antifouling, anti-corrosion, anti-icing, and self-cleaning applications [12,13,14,15,16,17]. These coatings are inspired by the unique wettability of plants and animals found in nature, such as lotus leaves [18], water striders’ legs [19], rose petals [15], etc. The application of superhydrophobic coatings in oil-contaminated environments is restricted since these coatings, even with basic micro- and nano-rough structures, are frequently unable to repel oil and other liquids with a surface energy lower than water [20,21]. In order to expand the coating’s wettability resistance from water to oil, the concept of superamphiphobic surfaces has emerged, i.e., the contact angle of both water and oil is greater than 150°, and the sliding angle of both water and oil is less than 10° [22,23,24]. and the generally accepted prerequisites for the development of superamphiphobic surfaces—that is, materials with extremely low surface energy and specifically designed surface structures [20], like kangaroo, overhanging [13], and re-entrant structures [25,26]—have been established. In order to create re-entrant structures on the surface of silicon wafers, Kang et al. [27] used lithography to produce mushroom-shaped micropillar arrays with great structural fidelity. It was discovered that these structures were super-liquid repellent to low surface energy liquids like white mineral oil. In order to create meter-like porous structured superamphiphobic coatings, Ganesh et al. [28] used PVA-TiO_2_ nano-fibers on glass substrates and the electrostatic spinning method. The prepared surfaces’ contact angles with ethylene glycol and water were 166° and 152.6°, respectively. Strict and exact control over the surface structure is necessary to construct such particularly formed superamphiphobic surfaces with high structural fidelity. This calls for the employment of labor-intensive and sophisticated processes like electrostatic spinning, photolithography, and reactive ion etching. These techniques have limited applicability, are frequently costly, and are dependent on the substrate material for the manufacture of superamphiphobic surfaces.

The most promising solution to this problem now available is the spraying method, which is easy to use, quick, affordable, and suitable for a wide range of large-area surfaces [29,30,31,32]. There is a dearth of literature on superamphiphobic coatings because the spray method builds up nano-particles on the coating’s surface, and it is challenging to control the coating’s morphology to meet the requirements of superamphiphobic microstructures [28,33,34]. In the literature that has been reported thus far, the coatings sprayed with nano-particle vegetation are primarily superhydrophobic coatings. This is because the requirements of superhydrophobic coatings on the surface morphology are relatively easy to meet. Wang [35] claims that, compared to nano-spherical particles with rough surfaces, the coating made by the spraying method using smooth nano-spheres has much less liquid repellency. Cassie’s principle [21] states that the solid-liquid contact area and contact angle decrease with increasing filler particle surface roughness. Consequently, employing the spraying method to create superamphiphobic coating microstructures might be aided by creating superamphiphobic filler particles with rough surfaces.

Prior to coating application, the coating must have a suitable balance of mechanical stability and liquid repellency [17,36]. In order to make the coatings adaptable to more demanding environments, the researchers tried to introduce different kinds of binders to enhance the mechanical durability of the coatings [31,37]. The superamphiphobic surfaces formed only by the agglomeration and accumulation of nano-superamphiphobic fillers are almost always fragile and have poor mechanical durability [38]. Natural wear and tear or even a slight touch may destroy their texture structure and lead to the loss of their superamphiphobicity [17,20,29]. To create a multilayer superamphiphobic covering, Zhu [30] used transparent silicone resin as a binder and sprayed micro-nano-particles on top of the silicone resin. Zhou [19] was able to create a coating that could sustain superamphiphobicity under extreme stretching conditions by using PDMS as a binder and spraying silicon nanofilaments onto a substrate that had already been stretched. The surface structure exhibits poor stability and robustness as the addition of binder increases the bonding force between the coating and the substrate. However, the nano-particle stacking causes a small inter-particle gap that restricts the entry of binder, and the attraction between the agglomerated nano-particles is weak, resulting in a poor inter-particle bonding force [39]. The in-situ growth of protective shells on the surface of their agglomerates can preserve the rough structure of the nano-particle-stacked structure and increase the robustness of the structure by utilizing the agglomeration effect of the nano-particles. This increases the stability of the entire coating to capture the air film.

In this work, the thermoplastic polymer polyvinylidene fluoride-hexafluoropropylene (PVDF-HFP), which has good binding strength and corrosion resistance, was chosen as the binder, and nano-spherical titanium dioxide particles were chosen as the precursor for the synthesis of 1H,1H,2H,2H-perfluorodecyltriethoxysilane (PFDTES) modified TiO_2_ (TiO_2_@fluoroPOS) functional fillers. Titanium dioxide rough armors were grown in situ on the surface of titanium dioxide agglomerates using the sol-gel method, utilizing the agglomeration effect of the nano-particles. Then, using 1H,1H,2H,2H-perfluorodecyltriethoxysilane (PFDTES), the TiO_2_ rough particles were fluorinated to produce TiO_2_@fluoroPOS fillers with a high fluorine content on the surface. This process produced functional fillers with low surface energy and high roughness. After mixing and spraying the filler and binder, a multifunctional P/TiO_2_@fluoroPOS superamphiphobic coating with outstanding stability was created. In terms of corrosion, self-cleaning, antifouling, anti-icing, and anti-waxing properties, the coating performed admirably.

## 2. Materials and Methods

### 2.1. Materials

Titanium dioxide (TiO_2_, spherical particles, particle size 20~30 nm) and polyvinylidene fluoride-hexafluoropropylene (PVDF-HFP, 99%) were supplied by Aladdin, Wallingford, CT, USA. Tetrabutyl titanate (TBT, AR, 98%), acetic acid (CH_3_COOH, 99.9%), 1H,1H,2H,2H-perfluorodecyltriethoxysilane (PFDTES, 97%), hydrochloric acid (HCl, AR, 36~38%), sodium hydroxide (NaOH, ACS, 97%), sodium chloride (NaCl, AR), and n-hexadecane (RG, 98%) were all supplied by Adamas, Emeryville, CA, USA. Anhydrous ethanol (EtOH, AR, 99.5%) and mineral oil (RG, 99%) were supplied by Greagent, Ringoes, NJ, USA. Soybean oil was supplied by Luhua, Yantai, China.

### 2.2. Preparation of TiO_2_@fluoroPOS Fillers

**Figure 1:**
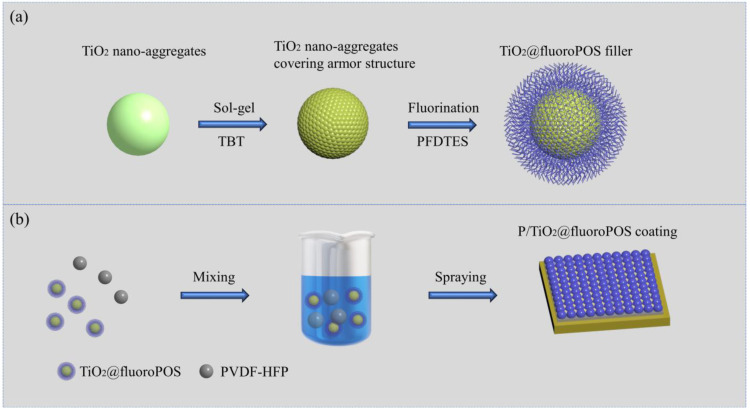
(**a**) The schematic illustration of the fabrication of filler TiO_2_@fluoroPOS. (**b**) The schematic illustration of the preparation of the superamphiphobic coating P/TiO_2_@fluoroPOS.

The TiO_2_@fluoroPOS superamphiphobic fillers were prepared by the sol-gel method, and the preparation process is shown in Figure 1a. Firstly, 2 g of TiO_2_ powder was dispersed in a mixed solution of 200 mL of anhydrous ethanol, 20 mL of deionized water, and 12 mL of acetic acid, then 6 mL of TBT was slowly added dropwise to the solution and stirred vigorously for 6 h to ensure complete hydrolysis. Subsequently, 1.6 mL of PFDTES was added dropwise to the solution and reacted for 12 h. Finally, the TiO_2_@fluoroPOS fillers were obtained after washing with anhydrous ethanol to a PH > 6.5 and drying at 70 °C for 12 h.

### 2.3. Preparation of P/TiO_2_@fluoroPOS Coatings

The coatings were prepared by spraying with a mixture of filler and binder, and the preparation process is shown in Figure 1b. The Al substrate was polished using 1000 mesh sandpaper to remove the oxide layer and then cleaned with deionized water and anhydrous ethanol. Next, 2 g of TiO_2_@fluoroPOS filler and 1.5 g of PVDF-HFP binder were dispersed in 20 mL of anhydrous ethanol. The dispersions were sprayed on the Al substrate (spraying area 24 cm^2^) using a spray gun at a distance of about 20 cm from the Al substrate and a pressure of 0.3 MPa. The filler surface concentration ranged from 41.7 to 62.5 g/m^2^. Finally, the superamphiphobic P/TiO_2_@fluoroPOS coating was obtained after curing at 220 °C for 60 min.

### 2.4. Characterization

The contact angles (CAs) of water and oil were measured by a contact angle measuring system (ZJ-6900, ZJ, Shenzhen, China). The sliding angles (SAs) of water and oil were measured by angular tilting platforms (GFWG60-60, MISUMI, Shanghai, China). The measurement results were the average value of the CAs and SAs at five positions with 10 μL water droplets and oil droplets. The crystal structures of the particles were determined by X-ray diffraction (XRD, X’Pert PRO MPD, Nalytical, Alemlo, The Netherlands). The functional groups of the original TiO_2_ and TiO_2_@fluoroPOS were analyzed by Fourier transform infrared spectroscopy (FT-IR, Nicolet iS 10, Green Bay, WI, USA). The surface chemistry of TiO_2_@fluoroPOS fillers was analyzed by X-ray photoelectron spectroscopy (XPS, Thermo Scientific K-Alpha, Waltham, MA, USA). Binding energies were calibrated with reference to the C1s peak at 284.8 eV. The surface morphology of the fillers and coatings was observed by scanning electron microscopy (SEM, Hitachi S3400N, Hitachi, Tokyo, Japan).

## 3. Results and Discussion

### 3.1. Analyses of TiO_2_@fluoroPOS Filler

**Figure 2:**
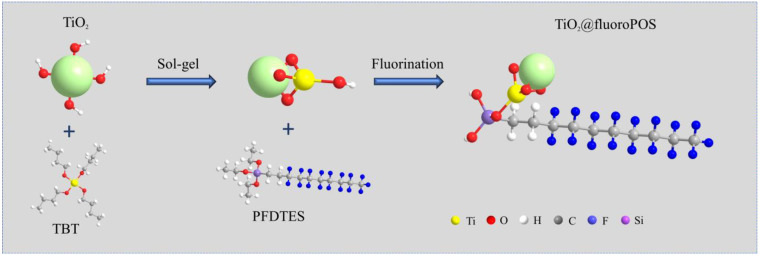
The reaction schematic for the fabrication of filler TiO_2_@fluoroPOS.

Figure 2 depicts the TiO_2_@fluoroPOS filler’s chemical reaction pathway. In an acidic environment, tetrabutyl titanate (TBT) readily hydrolyzes to form hydroxyl groups. Following a sequence of hydrolysis condensation reactions, TiO_2_ armors form on the surface of TiO_2_ agglomerates. The fluorine-containing groups of PFDTES are then grafted onto the surface of titanium dioxide via the condensation reaction, yielding a low-surface-energy functional filler.

**Figure 3:**
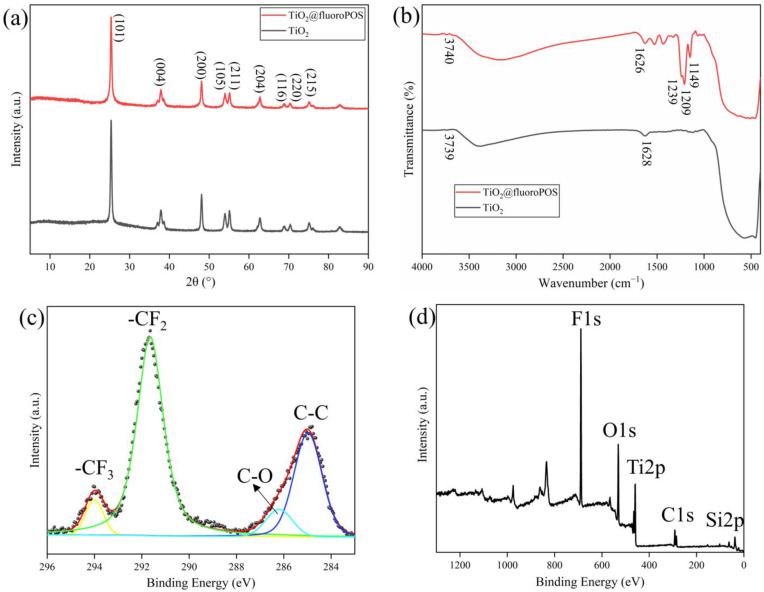
(**a**) XRD patterns of original TiO_2_ and TiO_2_@fluoroPOS; (**b**) FT-IR spectra of original TiO_2_ and TiO_2_@fluoroPOS; (**c**) XPS survey spectra of TiO_2_@fluoroPOS; (**d**) high-resolution C 1s spectra of TiO_2_@fluoroPOS.

Figure 3a displays the original TiO_2_ and TiO_2_@fluoroPOS XRD test results. Characteristic peaks of the original TiO_2_ are located at 2θ = 25.36°, 37.83°, 8.09°, 53.90°, 55.0°, 62.74°, 68.81°, 70.38°, and 75.12°, respectively; they correspond to the crystal planes (101), (004), (200), (105), (211), (204), (116), (220), and (215). The reacted TiO_2_@fluoroPOS characteristic peaks do not exhibit any discernible changes from the original TiO_2_, and they align with the standard peak base of anatase TiO_2_. This suggests that the TiO_2_ armors grown on the surface of the TiO_2_ agglomerates possess the same structure as the original TiO_2_.

Figure 3b displays the results of the Fourier transform infrared spectroscopy (FT-IR) measurements of the original TiO_2_ and TiO_2_@fluoroPOS. The primary cause of TiO_2_’s absorption peak at 3739 cm^−1^ is water from crystallization, while the vibration of immobilized hydroxyl groups on the surface of TiO_2_ is responsible for the absorption peak at 1628 cm^−1^. TiO_2_@fluoroPOS’s absorption peaks at 3740 cm^−1^ and 1626 cm^−1^ indicate the existence of hydroxyl groups and water crystallization. The three new absorption peaks at 1239 cm^−1^, 1209 cm^−1^, and 1149 cm^−1^ are due to the stretching vibrations of the -CF_3_ and -CF_2_ groups of PFDTES. This suggests that the groups containing fluorine have effectively branched onto the TiO_2_ armor surface.

As seen in Figure 3c,d, XPS was used to confirm the chemical composition of TiO_2_@fluoroPOS. The presence of many fluorine elements on the surface of the TiO_2_@fluoroPOS filler was indicated by the detection of a high peak of F1s at 689.05 eV. Furthermore, the -CF_3_, -CF_2_, C-O, and C-C groups of PFDTES were detected as the peaks at 294.0 eV, 291.7 eV, 286.2 eV, and 284.8 eV in the C1s high-energy spectra. In order to create low surface energy, which is essential for building the superamphiphobic coating, the results show that several low surface energy groups have been effectively branched onto the filler’s surface.

### 3.2. Surface Morphology of TiO_2_@fluoroPOS Fillers and P/TiO_2_@fluoroPOS Coatings

**Figure 4:**
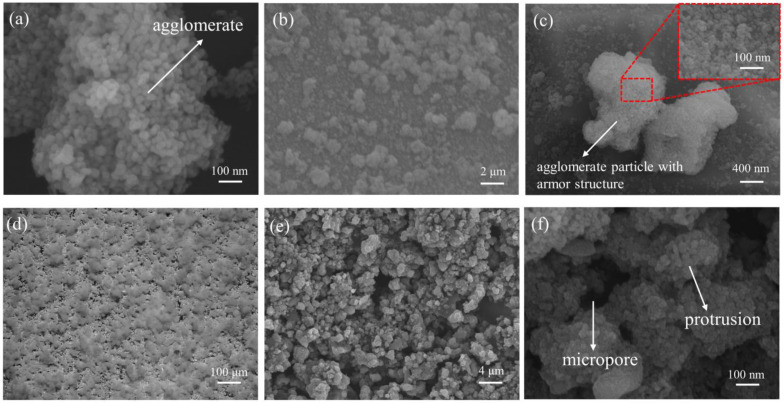
The SEM images of (**a**) TiO_2_ and (**b**,**c**) TiO_2_@fluoroPOS fillers. (**d**) Optical microscope photograph of P/TiO_2_@fluoroPOS coatings. (**e**,**f**) The SEM images of P/TiO_2_@fluoroPOS coatings.

Figure 4a displays the microstructures of the original TiO_2_. The surface of the untreated TiO_2_ is smooth and spherical, with individual particles having a diameter of roughly 20~30 nm. Van der Waals forces cause the TiO_2_ nano-particles to aggregate into agglomerates, which have a rough, uneven surface morphology. Despite this, van der Waals forces do not strongly bond the particles together, and there are clear interstitial gaps between the particles. TiO_2_@fluoroPOS’s microstructure can be observed in Figure 4b,c. Agglomerated particles with armor structure are roughly 1~2 μm in diameter. The surface of the particles retains the original rough structure of the agglomerates, and the granular protrusions are tightly bound to encapsulate the agglomerates. During the sol-gel process, the titanium dioxide armored structure grows in situ along the agglomerate surface, encapsulating the agglomerates into a micrometer particle with a surface nano-roughness structure that is critical to the robustness and construction of the superamphiphobic coatings.

The P/TiO_2_@fluoroPOS coating’s morphology is displayed in Figure 4d–f. The optical microscope image exhibits hilly bumps distributed on the surface of the coating. A solid and unique micro-nano roughness structure is formed by the fillers with a micro-nano roughness structure layered interleaved on the coating’s surface, creating a lot of protrusions and micropores. The fillers are adhered to and supported by the polymer binder. The coating is in the Cassie–Baxter state, which facilitates the suspension of droplets on top of the rough surface and results in superamphiphobicity. It does this by relying on the numerous papillae, micropores, and other small gaps working together to capture a stable air film.

### 3.3. Wettability of P/TiO_2_@fluoroPOS Coatings

**Figure 5:**
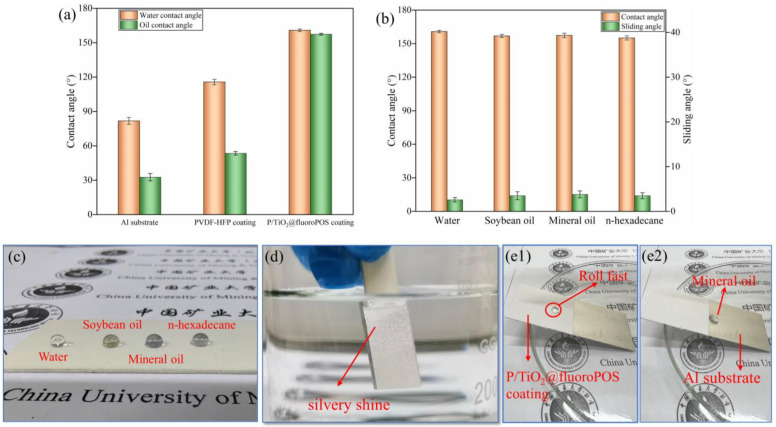
(**a**) Wettability of water and mineral oil on different samples. (**b**) The contact angle and sliding angle of various liquid droplets on P/TiO_2_@fluoroPOS coating. (**c**) Photograph of various liquid droplets on the P/TiO_2_@fluoroPOS coating. (**d**) The silver mirror effect. (**e1**,**e2**) Rolling of mineral oil on a substrate composed of P/TiO_2_@fluoroPOS and Al.

The wettability of the Al substrate, PVDF-HFP coating, and P/TiO_2_@fluoroPOS coating was assessed by measuring the contact angles of water and mineral oil. Figure 5a demonstrates that the naked Al surface is both hydrophilic and amphiphilic, but the PVDF-HFP surface is hydrophobic and lipophilic. The contact angles of the P/TiO_2_@fluoroPOS coating for water and mineral oil were 160.8° and 157.4°, respectively. This demonstrates the coating’s exceptional superamphiphobicity.

The wettability of the P/TiO_2_@fluoroPOS coatings was assessed by measuring the contact angles of water and mineral oil at various particle concentrations. The coatings maintained a filler-to-binder mass ratio of 4:3. The purpose was to investigate the impact of different particle concentrations on the wettability of the coatings and identify the critical concentration required to achieve superamphiphobic properties. Table 1 demonstrates a notable association between the concentration of particles and the hydrophobic and oleophobic characteristics of the coating. At a particle concentration of 6.19 g/m^2^, the coating attains superhydrophobicity. When the particle concentration increases to 20.69 g/m^2^, the oil contact angle of the coating measures 149.8°, which is near the critical concentration required for achieving superamphiphobicity. At a concentration of 29.97 g/m^2^, the coating exhibits exceptional superamphiphobic properties. Subsequently, when the concentration increases further, the contact angle reaches a plateau.

In order to evaluate the wettability of different surface tension (γ) liquids on the surface of the P/TiO_2_@fluoroPOS coating, the contact angle and sliding angle of various droplets, such as water (γ = 72.8 mN/m), soybean oil (γ = 33.8 mN/m), mineral oil (γ = 30.7 mN/m), and n-hexadecane (γ = 27.4 mN/m) [30,40], were measured. All of these liquids had contact angles larger than 150° and sliding angles smaller than 10°, as seen in Figure 5b. The contact angle of water was measured to be 160.8°, whereas the contact angle of n-hexadecane was found to be 155.1°, and the sliding angle was measured to be 3.5°. The P/TiO_2_@fluoroPOS coatings were coated with droplets of all four liquids, and, as shown in Figure 5c, the droplets were able to retain their spherical or ellipsoidal shapes. A silvery shine on the coating is observed when the coating is submerged in the liquid, as depicted in Figure 5d. This silvery shine was formed due to the total reflectance of light on the air film trapped on the surface. This specific event suggests that the coating’s surface can isolate the coating from the liquid by trapping and holding a stable air film. When mineral oil drops are applied to two different surfaces of aluminum plates positioned at an incline (P/TiO_2_@fluoroPOS coating, aluminum plate), as depicted in Figure 5(e1, e2), the droplets of mineral oil move quickly on the P/TiO_2_@fluoroPOS coating without sticking to it. However, when the coating comes into contact with the aluminum plate, the droplets spread out rapidly, and the movement becomes more fluid, but at a slower pace. Additionally, the droplets adhere to the coating, demonstrating exceptional performance. The velocity was significantly reduced, and there were adhered oil droplets. The coatings of P/TiO_2_@fluoroPOS exhibited exceptional superamphiphobic properties.

### 3.4. Corrosion Resistance of P/TiO_2_@fluoroPOS Coatings

**Figure 6:**
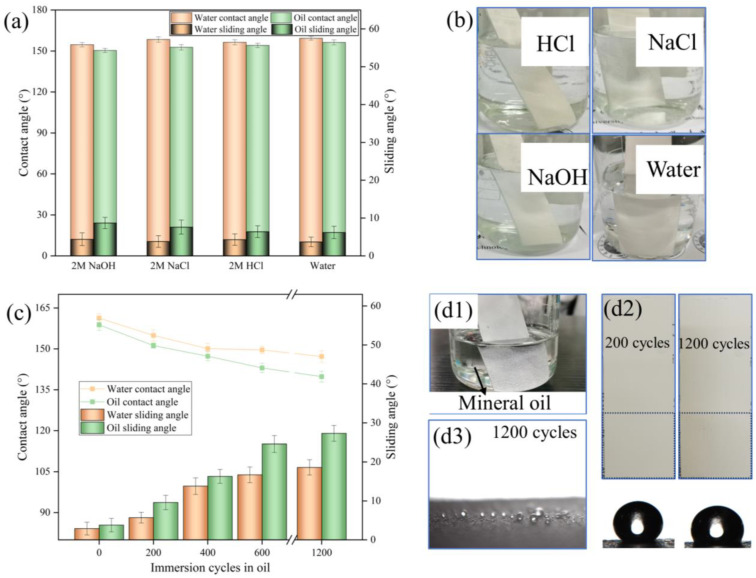
(**a**) Wettability of P/TiO_2_@fluoroPOS coating after immersion in 2M NaOH, 2M NaCl, 2M HCl, and water for 12 h. (**b**) Photograph of P/TiO_2_@fluoroPOS coating immersion in 2M NaOH, 2M NaCl, 2M HCl, and water. (**c**) Wettability of P/TiO_2_@fluoroPOS coating with immersion cycles in mineral oil. (**d1**) Photograph of P/TiO_2_@fluoroPOS coating immersion in mineral oil; (**d2**) photograph of the coating after 200 and 2000 cycles; (**d3**) droplets of oil sticking to the coating’s surface.

In order to evaluate the corrosion resistance and stability of the coatings in extreme external environments, they were immersed in solutions containing 2 mol/L of NaOH, HCl, and NaCl. Figure 6b demonstrates a clear silver mirror effect at the boundary between the solid and liquid. This indicates that the coating has successfully maintained a stable air layer in the corrosive solution environment. Following a 12-h immersion, the contact angle and sliding angle were determined. The results in Figure 6a revealed that the coating maintained a contact angle over 150° and a sliding angle below 10°, demonstrating exceptional superamphiphobic performance even after being exposed to the corrosive solution. The P/TiO_2_@fluoroPOS coatings exhibit exceptional superamphiphobic characteristics and demonstrate a strong aversion to corrosive solutions. According to the Cassie state, air may be easily trapped in the small gaps between the protrusions. This creates a barrier that makes it challenging for water and corrosive ions to access the surface of the coating. The repulsion hinders the wetting of the coating surface by the liquid, consequently decreasing the contact area between the liquid and the coated surface. Corrosion typically happens when a liquid comes into contact with a material’s surface and a chemical reaction occurs. By minimizing the contact area, corrosion can be effectively prevented.

### 3.5. Oil Repellency of P/TiO_2_@fluoroPOS Coatings

Superamphiphobic coatings often exhibit antifouling characteristics, but with limitations imposed on their applicability to ordinary liquids such as water and cooking oil. However, mineral oil has a low surface tension and a high adherence to the substrate or coating surface; thus, the coating’s resistance to adhesion must meet strict standards. To evaluate the durability of the coatings in an environment where they are immersed in mineral oil, specifically focusing on their superamphiphobic properties, the P/TiO_2_@fluoroPOS coatings were immersed in mineral oil and then extracted, with each instance being documented as a cycle (Figure 6(d1)). Following a certain number of cycles, the coatings were assessed for their contact angles and slide angles. As shown in Figure 6c, after being immersed for 200 cycles, the coating continues to exhibit superamphiphobicity. However, after 400 cycles of immersion, the oil contact angle (OCA) of the coating reduces to 147.3°. The contact angle exhibits a gradual reduction as the number of cycles increases. As the cycles approached 1200, the contact angle consistently maintained over 135°, indicating a continued ability to effectively repel liquids. After an extended period of immersion cycle testing, small spherical droplets will adhere to the surface of the coating (Figure 6(d2,d3)). This is likely due to the infiltration of mineral oil into the micro-nano protrusions on the coating surface, which increases the adhesion force. Consequently, the coating transitions from the Cassie state to a coexisting state of Wenzel and Cassie [41,42]. During each measurement, when the mineral oil comes into contact with the coating surface, some of the micro-nano protrusions may be re-infiltrated, displacing air and causing a change in the contact angle.

The inclusion of PVDF-HFP and TiO_2_@fluoroPOS fillers in the coating imparts it with low surface energy groups, resulting in an inert barrier and exceptional chemical resistance under challenging conditions, such as highly corrosive liquids and mineral oils. The combination of structural and compositional stability contributes to the overall stability of the collected gas film, ensuring that the coating offers versatile protection for the substrate in practical settings.

### 3.6. Anti-Icing of P/TiO_2_@fluoroPOS Coatings

**Figure 7:**
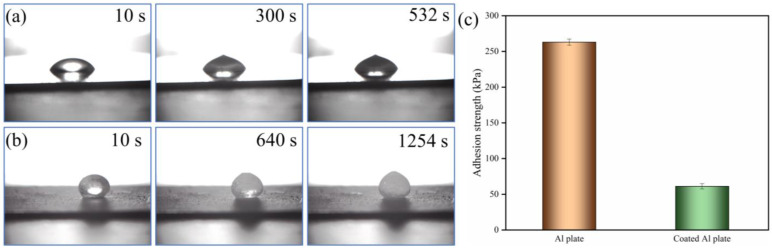
The freezing process of a water droplet (0.1 mL) at a temperature of −15 °C on the (**a**) Al plate and (**b**) P/TiO_2_@fluoroPOS coating; (**c**) Ice adhesion strength on the Al plate and P/TiO_2_@fluoroPOS coated Al plate at −15 °C.

Figure 7a,b illustrates the solidification of water on the Al plate’s surface and the P/TiO_2_@fluoroPOS coating at a temperature of −15 °C. The water droplets on the surface of the aluminum plate underwent full solidification after a duration of 530 s. By contrast, the water droplets on the P/TiO_2_@fluoroPOS coating took 1254 s to freeze. The freezing duration of the water droplets was noticeably prolonged when shielded by the P/TiO_2_@fluoroPOS coating. The inherent hydrophilicity of the uncoated aluminum plate facilitated the wetting of the coating surface by water droplets, leading to an increased contact area between the solid and liquid phases. As a consequence, heat transfer across the solid-liquid interface was accelerated at low temperatures. On the other hand, the shape of water droplets on the P/TiO_2_@fluoroPOS coating is spherical, and the coating maintains its superhydrophobic properties even at low temperatures. The coating’s surface is non-wetted, resulting in a significant decrease in the contact area between the solid and liquid. This results in a notable decrease in the rate at which heat is conducted, thereby causing a delay in the freezing of water droplets.

At a temperature of −15 °C, the ice adhesion strength on the surface of an Al plate is 263.1 kPa, whereas the ice adhesion strength on the surface of a P/TiO_2_@fluoroPOS coating is 61.2 kPa, as shown in Figure 7c. The ice adhesion strength on the coated surface is greatly reduced, facilitating the fast removal of ice from the surface. The coating may effectively prevent ice formation by delaying the freezing of water droplets and lowering the strength of ice adhesion, thereby achieving the objective of anti-icing.

### 3.7. Anti-Waxing of P/TiO_2_@fluoroPOS Coatings

**Figure 8:**
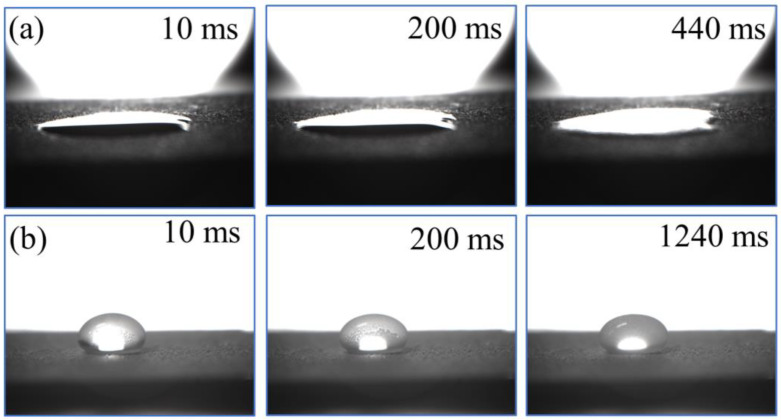
The waxing process of a paraffinic droplet (0.1 mL) at a temperature of 20 °C on the (**a**) carbon steel plate and (**b**) P/TiO_2_@fluoroPOS coating.

Wax accumulation issues in the petroleum industry may be resolved by using liquid-avoiding superamphiphobic coatings. Figure 8a,b illustrates the process of paraffin droplets forming wax on the surface of a carbon steel plate and P/TiO_2_@fluoroPOS coating at a temperature of 20 °C. The paraffin droplets on the carbon steel plate underwent quick infiltration and dispersion, followed by full solidification within 440 milliseconds, resulting in strong adhesion to the iron plate’s surface. On the other hand, the paraffin droplets present on the P/TiO_2_@fluoroPOS coating exhibited an ellipsoidal shape and took 1240 ms to fully solidify. This considerably prolonged the time it took for wax to form. The wax droplets were applied to the coating surface and exhibited an ellipsoidal morphology. The contact area between the paraffin wax and the coating was minimal, suggesting that the coating had a propensity to evade the wax droplets. It was postulated that this phenomenon could be attributed to the presence of an air film on the coating surface, acting as an anti-adhesion layer, which impeded the wetting of the paraffin wax droplets. Simultaneously, the limited thermal conductivity of air impedes the propagation of heat from the paraffin droplets to the frigid surface. The presence of an air film serves as a thermal barrier and anti-adhesion layer, thereby impeding the pace of wax bonding and diminishing the bonding strength. Consequently, this coating proves to be efficacious in preventing wax bonding and effectively addresses the issue of anti-waxing during the transportation of crude oil.

### 3.8. Self-Cleaning Ability of P/TiO_2_@fluoroPOS Coatings

**Figure 9:**
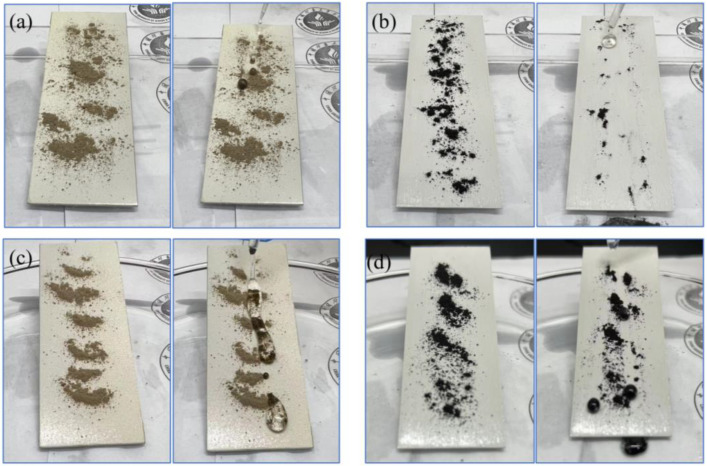
Self-cleaning process on the fabricated P/TiO_2_@fluoroPOS coating driven by (**a**,**b**) water and (**c**,**d**) mineral oil.

Water and mineral oil were used as driving droplets to evaluate the self-cleaning efficacy of P/TiO_2_@fluoroPOS coatings. As shown in Figure 9a–d, clays and coal were chosen as surface pollutants and equally distributed on the coated surface positioned at an incline. Drops of water and mineral oil were then introduced separately. The test findings indicated that the coating’s surface facilitated the rolling movement of water and oil droplets, enabling them to penetrate and remove impurities from the coating’s surface. The findings indicate the coating’s exceptional ability to self-clean its surface in both water and oil liquid conditions.

### 3.9. Mechanical Stability of P/TiO_2_@fluoroPOS Coatings

#### 3.9.1. Water Jetting Test

**Figure 10:**
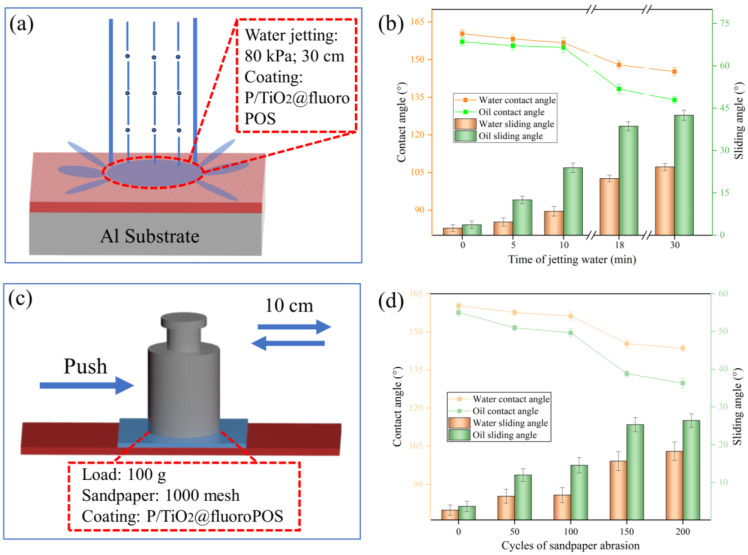
(**a**) Schematic of sandpaper abrasion; (**b**) the wettability of P/TiO_2_@fluoroPOS coating after sandpaper abrasion. (**c**) Schematic of the water jetting test; (**d**) the wettability of the P/TiO_2_@fluoroPOS coating after water jetting.

Given the environmental conditions in which superamphiphobic coatings are used, fluid jetting is often inevitable. Water jetting experiments were performed to evaluate the impact resistance of the P/TiO_2_@fluoroPOS coating. The coating was positioned in a horizontal orientation, 30 cm below the water flow, at a pressure of 80 kPa, as seen in Figure 10a. The contact angle and sliding angle of the coating were measured after a certain duration of water impact. The findings may be shown in Figure 10b. Following a 10-min water jetting process, the contact angle of the coating did not exhibit a substantial decline and remained over 150°. However, the sliding angle experienced a large rise, reaching x°. The armor structure of the TiO_2_@fluoroPOS fillers successfully withstood the forceful flow of water, preventing quick degradation of the nano-structure of the coating and ultimately avoiding coating failure. During the time period of 10~18 min, when water is sprayed onto the coating, the contact angle decreases at a faster rate. This leads to a loss of superamphiphobicity, and some of the rough structure that is not strongly bonded with the binder is damaged. After 18 min of exposure to the water flow, the contact angle decreases at a slower rate and eventually stabilizes. Following a 30 min impact, the contact angle of the coating remained consistently over 130°.

#### 3.9.2. Sandpaper Abrasion Test

One of the main obstacles to the widespread use of superamphiphobic coatings is their surface structure’s fragility. Sandpaper abrasion tests were used to assess the mechanical resilience of the P/TiO_2_@fluoroPOS coatings that were produced. As shown in Figure 10c, square sandpaper was positioned squarely against the coating (contact area 16 cm^2^) and moved 10 cm in a single direction while bearing a 100 g load set as a single cycle. Figure 10d displays the test findings. After 100 abrasion cycles, the coating’s superamphiphobicity did not alter. After 200 cycles, however, a portion of the coating’s surface structure was damaged, and its oca was still larger than 130°, maintaining strong liquid repellency. The nanoclusters attain a micron-scale size subsequent to the development of protective armor on their surface. The micron particles undergo a secondary rough structure on the surface, resulting in a nano-meter scale structure. The binder secures the micron particles to withstand tangential forces, while the surface armor safeguards the nano-particles from damage to the nano-structure. The combined effect of the binder and protective armor ensures exceptional mechanical stability for the coating.

### 3.10. Superamphiphobicity Mechanism

**Figure 11:**
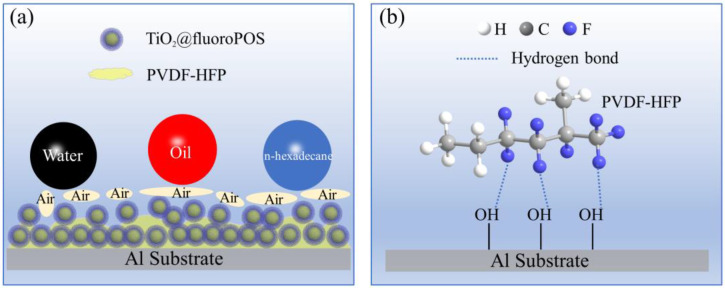
(**a**) Schematic diagram of the mechanisms of air film stability on the P/TiO_2_@fluoroPOS coating surface. (**b**) Hydrogen bond interactions between the Al substrate and PVDF-HFP.

PVDF-HFP and TiO_2_@fluoroPOS fillers enhanced with low surface energy groups—which have outstanding liquid repellency, corrosion resistance, and mechanical stability—make up the P/TiO_2_@fluoroPOS superamphiphobic coating. As seen in Figure 11a, a potential mechanism for the interaction between the coating and liquid was suggested in order to clarify the superamphiphobicity mechanism of the coating. Many micro-convex and inwardly concave microporous structures were formed by the accumulation of fillers on the coating surface. By using this structure and the low surface energy TiO_2_@fluoroPOS fillers, an air film could be captured on the coating’s surface. The nano-microstructures on the coating’s surface are a part of the fillers’ rough armor, which is difficult to destroy. The rough armor is fixed by the PVDF-HFP connection between the encapsulated agglomerates, ensuring stability for both the structure and the captured air film. The Cassie–Baxter equation [17,21,43] states that cosθ_D_ = f_s_cosθ_Y_ − f_g_, where θ_D_ represents the apparent contact angle and θ_Y_ represents the liquid-solid intrinsic contact angle. In the ideal Cassie state, the sum of f_s_ and f_g_ (liquid-solid/gas-solid contact fractions) is 1, resulting in cosθ_D_ = f_s_(1 + cosθ_Y_) − 1. Therefore, as the liquid-solid contact fraction fs decreases, the apparent contact angle θ_D_ significantly increases, leading to the repulsion of low surface energy droplets.

PVDF-HFP serves as a binding agent in the coating process, facilitating improved interparticle bonding and promoting robust adhesion to the coating. This enables a secure link between the substrate and the coating, thereby mitigating tangential friction. Figure 11b illustrates the hydrogen bonding interactions between the F atoms in the PVDF-HFP and the -OH groups on the Al substrate.

## 4. Conclusions

In this study, a novel superamphiphobic coating consisting of P/TiO_2_@fluoroPOS was effectively synthesized using a straightforward and scalable spraying method. The coating exhibited corrosion resistance, self-cleaning properties, anti-fouling capabilities, anti-ice-covering properties, and anti-waxing properties. The superamphiphobic filler with micro-nano structure was obtained by growing rough armors on the surface of titanium dioxide agglomerates using the sol-gel process and subsequently fluorinating them. The coatings constructed using PVDF-HFP and TiO_2_@fluoroPOS fillers exhibit highly stable air films due to the synergistic effects of filler structural stabilization and binder immobilization.

Many liquids with varying surface tensions exhibit repulsion towards the coating, including n-hexadecane (contact angle: 155.1°, sliding angle: 3.5°) and water (160.8°, sliding angle: 2.6°). Furthermore, the coating exhibits remarkable mechanical and chemical stability, and it retains its superamphiphobicity even after being immersed for 12 h in solutions containing 2 mol/L HCl, NaOH, and NaCl. In addition, the coating oil contact angle retains more than 130° even after 200 cycles of sandpaper abrasion and 30 min of 80 kPa water jetting. The coating also performs exceptionally well in terms of self-cleaning, anti-icing, and anti-waxing. With a wide range of potential applications in real-world industrial production, the current work offers a novel concept for immobilizing nano-particles on the coating’s surface to improve its mechanical and chemical resilience.

## Figures and Tables

**Table 1:** Influence of surface particle concentration on the liquid repellency of coatings.

Concentration of Particles (g/m^2^)	Water Contact Angle (°)	Standard Deviation	Oil Contact Angle (°)	Standard Deviation
6.19	154.4	1.30115	136.3	2.15244
11.75	157.6	1.69794	144.1	2.61381
20.69	159.5	1.39104	149.8	1.56301
29.97	160.2	1.01833	154.8	1.57575
36.36	160.7	1.23369	157.2	1.10317

## Data Availability

The raw data supporting the conclusions of this article will be made available by the authors on request.
